# Retinal Prosthetic Approaches to Enhance Visual Perception for Blind Patients

**DOI:** 10.3390/mi11050535

**Published:** 2020-05-24

**Authors:** Shinyong Shim, Kyungsik Eom, Joonsoo Jeong, Sung June Kim

**Affiliations:** 1 Department of Electrical and Computer Engineering, College of Engineering, Seoul National University, Seoul 08826, Korea; simsinyong@gmail.com; 2 Inter-university Semiconductor Research Center, College of Engineering, Seoul National University, Seoul 08826, Korea; 3 Department of Electronics Engineering, College of Engineering, Pusan National University, Busan 46241, Korea; 4 School of Biomedical Convergence Engineering, College of Information and Biomedical Engineering, Pusan National University, Yangsan 50612, Korea; 5 Institute on Aging, College of Medicine, Seoul National University, Seoul 08826, Korea

**Keywords:** retinal prosthesis, vision restoration, visual perception, retinal stimulation, high resolution

## Abstract

Retinal prostheses are implantable devices that aim to restore the vision of blind patients suffering from retinal degeneration, mainly by artificially stimulating the remaining retinal neurons. Some retinal prostheses have successfully reached the stage of clinical trials; however, these devices can only restore vision partially and remain insufficient to enable patients to conduct everyday life independently. The visual acuity of the artificial vision is limited by various factors from both engineering and physiological perspectives. To overcome those issues and further enhance the visual resolution of retinal prostheses, a variety of retinal prosthetic approaches have been proposed, based on optimization of the geometries of electrode arrays and stimulation pulse parameters. Other retinal stimulation modalities such as optics, ultrasound, and magnetics have also been utilized to address the limitations in conventional electrical stimulation. Although none of these approaches have been clinically proven to fully restore the function of a degenerated retina, the extensive efforts made in this field have demonstrated a series of encouraging findings for the next generation of retinal prostheses, and these could potentially enhance the visual acuity of retinal prostheses. In this article, a comprehensive and up-to-date overview of retinal prosthetic strategies is provided, with a specific focus on a quantitative assessment of visual acuity results from various retinal stimulation technologies. The aim is to highlight future directions toward high-resolution retinal prostheses.

## 1. Introduction

The retina is a thin layer of light-sensitive tissue that lies at the back of the eye, and it is the innermost layer of the eye [1]. Light passes through a series of optical elements in the eye, e.g., the cornea, iris, lens, and vitreous humor. The light is then focused onto photoreceptors in the retina, which convert the incident light into electrical neural signals. The neural impulses are processed while passing through retinal layers including bipolar cells, horizontal cells, amacrine cells, and ganglion cells, before travelling via the optic nerve to the visual cortex, where we ultimately perceive the visual information [2].

Retinitis pigmentosa (RP) and age-related macular degeneration (AMD) are the two most prevalent retinal degenerative diseases. They affect millions of individuals worldwide and ultimately cause permanent blindness, owing to the loss of photoreceptors [3,4,5]. RP comprises a group of genetically inherited blindness disorders indicated by the functional loss of photoreceptors in the retina, estimated to affect roughly one in 4000 people both in the United States and worldwide [6,7]. Its symptoms include tunnel vision and decreased night vision, both of which result from the degeneration of rod cells in the peripheral retina [7,8,9]. This gradual loss of the peripheral vision can also trigger damage to cone cells in the macula and other retinal cells, exacerbating the degree of blindness. AMD is the progressive deterioration of the central vision primarily affecting the cones, and it eventually leads to blurred vision or complete blindness [10,11,12]. AMD is prevalent in the elderly population, affecting more than two million people in the United States, and 30–50 million people worldwide [13,14,15].

Although current remedies can slow the progression of vision loss, there exist no permanent cures for either RP or AMD for now [16,17]. Extensive efforts have been devoted to restoring the resultant impaired visual function, based mainly on gene therapy, stem cell transplantation, or retinal prostheses [18,19,20,21,22,23,24,25]. Gene therapy has shown sustained improvements in the visual function of dogs suffering from RP [21,26]. Although some gene therapy methods have been translated to clinical trials with a primary focus on evaluation of their safety [23,24], it is presently considered too immature to provide clinical solutions for visually impaired patients, as only a limited number of mutations associated with these diseases have been recognized. Stem cell transplantation has also been broadly investigated, e.g., to determine whether stem cells can differentiate into specific retinal neurons and replace the function of photoreceptors in animal AMD models [19,20,27,28]. Nevertheless, a more in-depth understanding is required before clinical applications are explored, to address the discrepancies between patients suffering from AMD and the available animal models in terms of the stages of initiation and the progression of retinal degeneration [19].

Retinal prostheses are primarily based on electrical stimulation and have relatively rapidly reached clinical stages. They are presently acknowledged as the most viable technology for the treatment of RP and AMD [29,30]. Despite the photoreceptor degeneration and following reorganization of retinal structures in the cases of RP and AMD, it has been found that the inner retinal layers, including bipolar and ganglion cells, mostly retain their original morphology and functionality [31,32,33,34]. This preservation of the degenerated retina has enabled electrical stimulation (ES) to be feasibly employed for vision restoration via patterned activation of the remaining retinal cells, in correspondence with an image seen by the eyes. This approach can replace the function of photoreceptors. Several clinical trials have shown that blind patients with retinal prostheses can successfully perform several basic tasks, such as light localization, pattern discrimination, and reading characters [35,36,37].

**Figure 1:**
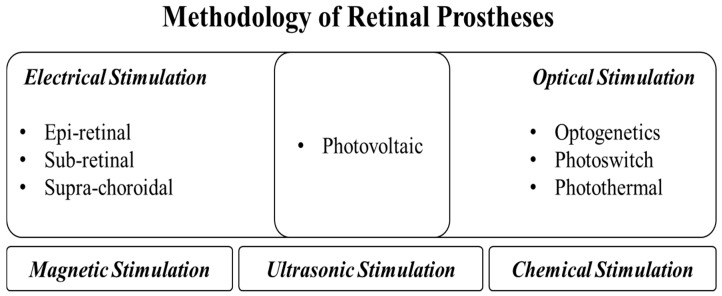
A summary of retinal prosthetic approaches for enhanced visual resolution.

Despite these promising results from the clinical trials in the early stages, there remains substantial room for further improvement, from both engineering and physiological perspectives. The most critical issue is the relatively low quality of the visual perception enabled by retinal prostheses when compared to normal vision. This is presumably attributed to the low spatial resolution of the stimulation in a dense network of retinal cells [5,25,38,39]. To achieve higher spatial resolution, a variety of retinal prosthetic strategies have been proposed for ES, based on special structures of electrodes or optimization of stimulation parameters. Moreover, strategies based on other modalities have been proposed, such as optogenetics, light-driven exogenous materials, ultrasonic waves, and magnetic fields, etc., as shown in Figure 1. This review aims to provide a detailed description of distinctive stimulation strategies, with a specific focus on a quantitative estimation of the spatial resolution. A discussion of future perspectives towards improvement of visual perception in visually impaired patients is also provided.

## 2. Electrical Stimulation (ES)

The ES of the nervous system has long been successfully achieved in various implantable devices, including pacemakers, deep-brain stimulators, cochlear implants, and retinal prostheses, for restoring impaired neural functions [40,41,42,43,44,45,46]. Vision restoration dates back to 1775, when Charles Le Roy applied an electric current around the head of a visually impaired patient to elicit light sensations [25]. In 1968, Brindley and Lewin showed that ES on the visual cortex could enable visual perception, later termed as “phosphenes” [47]. In 1969, Potts and Inoue reported that electrically evoked cortical responses in the visual system of RP patients were similar to visually elicited responses in normally sighted subjects [48]. Since the 1970s, rapid advancements in microfabrication technologies and surgical techniques have promoted a number of emerging studies towards the common goal of curing profound vision loss via various stimulation approaches, including the retina, optic nerve, or visual cortex [49,50,51,52,53,54,55,56].

Among the methods aimed at stimulation of the visual pathway, retinal stimulation has been studied the most extensively, owing to its advantages in retinotopic mapping, relatively lower surgical risks, and easier accessibility [57,58,59,60]. Several retinal prosthetic devices (such as the Argus II and the Alpha IMS) have been medically approved, with promising outcomes verified in clinical trials [25,37,61,62].

### 2.1. Classification of ES on the Retina

Retinal prostheses typically consist of multiple functional components, including image sensors, image processors, electrical stimulators, and electrode arrays. An image sensor captures real-time images from visual scenes to be translated into ES patterns. One of the most common image acquisition methods is to use an external camera mounted on a pair of glasses [62,63,64,65]. An ES pattern computed from a captured image is then transmitted into an implanted stimulator via radio frequency (RF) telemetry. Another image-capturing system employs photodiode arrays directly implanted in the retinal space [65,66]. Such silicon-based photodiode arrays can be natively integrated with a stimulator circuit to provide high scalability regarding the number of stimulating channels. Patterned ES (generated from either the implanted stimulator circuit or photodiode arrays) is then delivered to the retinal electrode array implanted in the retinal space, delivering electrical stimuli to the retinal cells for the patterned activation of the retinal neurons.

In general, ES-based retinal prostheses are classified into three types, depending on the location within the retinal layers where the electrodes are implanted: epi-retinal, sub-retinal, and supra-choroidal approaches. Outcomes from the respective studies that have been applied in clinical trials are summarized in Table 1.

#### 2.1.1. Epi-Retinal Prostheses

In the epi-retinal approach, electrodes are anchored on the inner surface of the retina, in the near vicinity of retinal ganglion cells (RGCs). The large volume in the vitreous humor makes the epi-retinal region relatively easy to access, which can reduce the risk of retinal damage during surgery. In addition, it has been reported that the heat generated from the retinal electrode arrays effectively diffuses into the vitreous cavity, alleviating concerns regarding potential thermal damage to tissue [78]. However, as the electrodes placed on the top of the retina directly activate RGCs, epi-retinal stimulation cannot exploit the native signal-processing mechanisms occurring in the retinal network. Nevertheless, epi-retinal stimulation has remained among the key targets of retinal prostheses.

The Argus II (Second Sight Medical Products, Inc., California, United States) was the first retinal prosthesis to receive approvals from both the European Union (CE Mark) in 2011 and the United States Food and Drug Administration (FDA) in 2013. The Argus II system comprises a camera mounted on glasses, external processing unit worn by a user, implantable unit consisting of stimulation electronics, and electrode array. The electrode array consists of 60 electrode channels, each with a diameter of 200 μm and a center-to-center separation of 575 μm. Compared to the previous model with 16 electrodes (Argus I), the Argus II provides improvements in terms of higher spatial resolution and larger coverage area, with a visual field of approximately 11° × 19° [67]. Clinical trials previously evaluated 30 subjects implanted with the Argus II in the United States and Europe, to study its safety and effectiveness regarding performing of real-world tasks [68,79]. With reference to the minimum angle of resolution (MAR), the average grating visual acuity was shown to be 2.5 logMAR (20/6325), and the highest acuity was shown to be 1.8 logMAR (20/1262) [67,68]. Additionally, most subjects could successfully perform several basic tasks, such as those concerning grating visual acuity, square localization, and movement detection [62,68,80,81,82]. Other evaluation methods reported from 2013 to 2017 have demonstrated the feasibility of the Argus II for vision restoration in RP patients, including tasks such as reading letters, grasping objects, sorting socks, sidewalk tracking, and discriminating walking directions [83,84,85,86]. Nevertheless, this group announced the winding down of operations as well as discontinued production of the Argus II and has adapted the retinal device for use as a cortical implant [87,88].

The retinal prosthesis developed by Intelligent Medical Implants (IMI) operates in a manner similar to that of the Argus II [70]. One difference is that the IMI employs an infrared (IR) optical link for data transmission, which enables a high data rate to be achieved, as well as a natural interruption of the “seeing” in accordance with eye closing or blinking. The epi-retinal electrode array of the IMI carries 49 electrodes, each with a diameter of 250 μm and a spacing of 120 μm. An acute study demonstrated that the IMI was effective in eliciting visual perception in 19 out of 20 RP subjects [70]. Furthermore, longer implantation in seven patients showed that the patients could distinguish simple patterns generated by the retinal stimulation with long-term safety for up to 30 months, without showing any sign of vascular leakage or tissue damage caused by the implant [69,71,72]. After its acquisition by the Pixium Vision in 2007, the device has evolved into the Intelligent Retinal Implant System (IRIS), featuring 150 electrodes for high-resolution stimulation and a smart event-based camera for capturing temporal changes of visual scenes [73,89]. In spite of the clinical approval of the CE Mark in 2016 and subsequent clinical trials to assess the safety and performance [73], the Pixium Vision has discontinued the development of the IRIS. Instead, they are focusing on a new retinal prosthesis based on photovoltaic silicon materials, named as the PRIMA, which will be discussed in Section 3.1.2 [90].

The EPI-RET3 system (from the Epi-Ret in Germany) exhibits a unique feature in that the electronics package is located in the posterior chamber of the eye. This enables total implantation of the device within the intraocular space, without leaving any transscleral cables or additional components attached to an eyeball. This concept can significantly reduce the risk of infections around the transscleral incision, mechanical stress to the package, and discomfort of the patients [74]. The 25-channel electrode array is arranged in a hexagonal shape, with a diameter of 100 μm and a pitch of 500 μm [35,74]. Each electrode site protrudes 25 μm toward the retina for higher proximity to the targeted neurons. These were able to successfully elicit visual sensations with a lower ES threshold in six RP patients in 2006 for four weeks [35]. In that study, the subjects were able to discriminate simple patterns, such as circles or lines. Future directions of the EPI-RET3 include the development of very large electrode arrays for covering up to 37° of the visual field [25,91].

#### 2.1.2. Sub-Retinal Prostheses

Sub-retinal electrode arrays are implanted behind a degenerated retina. The current pulses are injected into the outer and middle sections of the retina, exploiting the residual retinal processing network, while avoiding direct stimulation to RGCs which might activate unwanted axon fibers. The confined sub-retinal space places an anatomical constraint on the size of the array but simultaneously allows the array to be held by pressure, without any extra need for fixation.

The Alpha IMS (Retina Implant AG, Germany) was the first sub-retinal prosthesis approved by the CE Mark in 2013. It employs a micro-photodiode array (MPDA) for light detection but leverages a powering link from outside the body (via a wireless RF inductive link behind the ear) to amplify the MPDA-generated electrical currents [37,92,93]. The MPDA contains 1500 independently operating cells, each comprising a micro-photodiode, an amplifier, and a 50 μm × 50 μm electrode. Each micro-photodiode generates current pulses in response to the intensity of incident light to create 38-by-40-pixel images, with the amplitude distribution corresponding to the image projected onto the retina. The MPDA has an overall size of 3 mm × 3 mm and covers a visual field of approximately 11° × 11°. Interim clinical trials for the Alpha IMS between 2010 and 2014 assessed the visual acuity and object recognition of the restored vision in the context of daily living and mobility [66]. Among 29 patients, 25 patients reported light sensations, and 21 patients showed significant improvements in recognition tasks within a year of implantation. Three patients were able to read letters with a visual angle between 5° and 10°, demonstrating the highest visual acuity of 20/546 in a Landolt-C test. The next-generation device (Alpha AMS) contained 1600 channels and received CE approval in 2016. The one-year clinical results reported from 15 patients in 2017 showed functional benefits and safety profiles similar to those of the Alpha IMS [75]. The theoretical Snellen visual acuity of the Alpha AMS, approximately 6/60 (~20/200), has not yet been reported in any participants [76]. The Retinal Implant AG announced discontinuing business activities in 2019, while their research work will be continued in university environments [87,94].

The Boston Retinal Implant Project is one of the first endeavors in this field to lead acute trials in human subjects. In those trials, reproducible visual sensations via retinal stimulation were reported by patients with end-stage RP [95]. The system structure is similar to that of the Argus II; however, the Boston group developed a minimally invasive surgical technique for sub-retinal implantation of the electrode array, with minimized disruption to the retina [63,96]. First-generation devices with 15 electrodes were implanted in two minipigs, and postoperative examinations reported the continued functioning of these devices for up to five and a half months [97]. The device has advanced from containing 15 electrodes to the newest prototype containing 256 electrodes, currently in preclinical testing [98].

#### 2.1.3. Supra-Choroidal Prostheses

A supra-choroidal prosthesis is characterized by electrodes implanted between the choroid and the sclera, relatively distant from the retina as compared to its epi-retinal and sub-retinal counterparts. Separation of the device from the retina may reduce the risk of retinal damage during surgery. However, the longer current path from the electrodes to the retina may cause increased stimulation thresholds and the spread of electric charges, leading to low spatial resolution [99].

The Bionic Vision Australia (BVA) team has developed several prototypes of supra-choroidal prostheses. One prototype employs 33 stimulation electrodes: 30 electrodes with a diameter of 600 μm and three electrodes with a diameter of 400 μm [77,100]. Postoperative monitoring for two years in three RP patients in 2012 demonstrated that the devices remained stable and functional without causing significant retinal damage. Phosphenes could be elicited in all the patients, with an estimated average Landolt-C acuity of 2.62 logMAR (20/8397), and the highest acuity of 20/4451 [77]. The BVA team has also developed the next-generation 44-electrode device to provide a wider field of view [101,102]. In preclinical tests for safety evaluation in 2018, the electrode arrays implanted in 10 normal-sighted felines showed good tolerance during and after surgery, in addition to good conformability to the eye curvature for more than 13 weeks of the mean implantation period [102].

The semichronic suprachoroid transscleral prosthesis, developed by Fujikado et al. in Japan, contains a 49-channel array, with an individual diameter of 500 μm and spacing of 700 μm. In clinical trials of two RP patients in 2011, both patients were able to localize and discriminate white boxes; one patient could execute simple grasping tasks with higher accuracy than chance [36]. In subsequent trials with three additional subjects, one subject was able to identify squares better than patients without the device. Two subjects were even able to walk along a white line and recognize everyday objects [103,104].

### 2.2. Factors Limiting Spatial Resolution of ES

Several studies worldwide have demonstrated that ES-based retinal prostheses can restore partial vision in visually impaired individuals. The performance has been evaluated in patients suffering from RP or AMD in terms of light localization, movement detection, and grating discrimination, as well as in regard to real-world functional tasks including reading letters, detecting objects, and grasping objects. However, owing to technical and physiological challenges, ES-based retinal prostheses have yet to provide sufficient visual acuity to allow blind patients to lead independent lives.

The spatial resolution of retinal prostheses is limited by many factors, including density, geometries, channel layout, and return configuration of electrode arrays. Other aspects including pulse types and parameters, current spreading, inter-channel interference, imperfect retinotopic mapping, and inter-retinal signal processing associated with the target retinal cell types also significantly affect the visual resolution. The highest visual acuity achieved by ES-based retinal prostheses so far is 20/546 with the Alpha IMS in a Landolt-C test and 20/1262 with the Argus II in a grating acuity test. Both values are far lower than the theoretical visual acuity of the Alpha IMS, with 1500 electrodes with 70-μm spacing, and the Argus II, with 60 channels of 575 μm in diameter [105]. Notably, the practical visual acuity achieved by the Alpha IMS is not significantly higher than that of the Argus II, given its 25-fold higher number of stimulating channels. These results may imply that the patterned stimulation generated from all the 1500 individual channels of the Alpha IMS could not be perfectly discriminated by the retinal cells, lowering the perceived spatial resolution. It can also be inferred that simply increasing the density of stimulating channels would not necessarily achieve higher visual acuity in retinal prostheses. Even if a retinal electrode array with a channel size and density comparable to a single photoreceptor could be fabricated, it would not be able to reach the visual acuity of normal vision if the aforementioned factors limiting the visual acuity of retinal stimulation were not addressed.

### 2.3. Efforts to Improve Visual Acuity Using ES

To overcome those limitations, extensive efforts have been devoted to modifying structures of electrodes and optimizing ES parameters, as will be discussed in the following sections.

#### 2.3.1. Structural Modification of Electrodes

**Figure 2:**
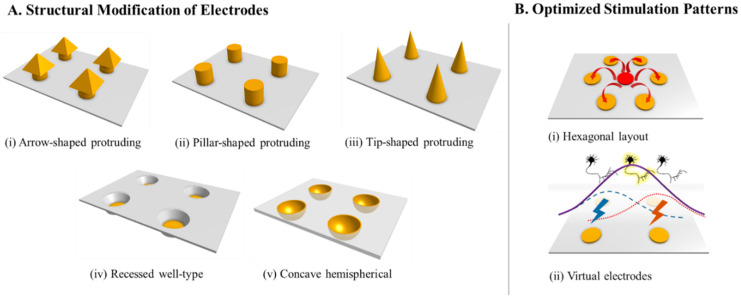
Schematic illustration of (**A**) featured retinal electrode structures for higher visual resolution including arrow-shaped, pillar-shaped, tip-shaped, recessed well-type, and concave hemispherical, and (**B**) optimized stimulation patterns based on hexagonal layout and virtual electrodes for current focusing and steering. (Adapted from [106,107,108,112,113,121,122]).

The spatial resolution of ES depends considerably on physical structures of the electrodes interfacing with the retina. These govern the distance to the targeted neurons, as well as the pattern of current spreading. One approach to addressing this issue is to create three-dimensional (3D) electrodes with protruding or recessed structures, as depicted in Figure 2A. In 2009, Koo et al. presented an 199 arrow-shaped 3D electrode array (Figure 2A (i)) with varying heights of 50–150 μm [106]. The electrode array had a potential to penetrate the inner limiting membrane of the retina to more effectively stimulate the degenerated retinal cells, and higher electrically evoked cortical potential was recorded in vivo using the 3D electrodes in comparison to conventional planar electrodes. In the same year, protruding pillar-shaped electrodes for sub-retinal prostheses were demonstrated, and closer proximity to the inner retinal cells was achieved than that achieved by planar electrodes with similar dimensions [107]. The pillar structure of 10 μm in diameter and 65 μm in height (Figure 2A (ii)) induced the minimal alteration of the inner retinal structure after 6 weeks following implantation, suggesting a promising approach for maintaining proximity to the target neurons. In 2010, Wang et al. demonstrated polymer-based 3D tip-shaped electrodes (Figure 2A (iii)) with a diameter of 100 μm and a height of approximately 80 μm. These electrodes could lower the electrode impedance to 72% of that of a flat electrode with the same footprint, as confirmed both in vitro and in vivo [108]. The similar shape of 3D penetrating electrodes was also leveraged by the Nano Retina group in Israel to demonstrate a lower charge threshold of less than 0.5 nC for evoking retinal responses [109,110]. On the other hand, in 2011, histological analysis revealed the migration of retinal cells into the cavities of a recessed well-like structure, whereby tight contact was achieved between the electrodes and target retinal cells, and neural interfaces with higher selectivity were provided in vivo [111]. This phenomenon was also demonstrated in vivo by Bendali et al. in 2015, i.e., the potential of a 3D well-shaped electrode (Figure 2A (iv)) could enhance the spatial resolution of retinal stimulation [112]. In addition to the cell contact, further analysis of the 3D structures through finite element simulation suggested that concave hemispherical electrodes (Figure 2A (v)) would be the ideal option owing to their superior stimulation localization capability, as they activated an RGC area less than half the size of the area activated by convex electrodes when the same current was injected [113].

#### 2.3.2. Optimization of Stimulation Patterns

Another approach for achieving higher spatial resolution is based on optimization of stimulation patterns to provide focused or steered retinal activation, for example, by varying the return electrode layout or stimulation levels between two or more adjacent electrodes, as shown in Figure 2B [114,115]. These strategies can confine the electric field within a narrower area for highly focused stimulation, as well as can steer the current path to create virtual electrodes for higher effective channel density. In 2005, Palanker et al. studied the effects of a common return electrode located on an elevated surface above electrode sites on the restriction of current spreading within well-like cavities, with the goal of providing more selective and localized stimulation [116]. A hexagonal electrode configuration of retinal electrode arrays (Figure 2B (i)), in which one stimulation electrode is surrounded by six return electrodes, has also proven effective in localized retinal stimulation, by lowering the stimulation threshold within the hexagonal array to a greater extent than that outside the hexagon [117]. This local threshold difference in the hexapolar stimulation enables the selective activation of only the neurons within the hexagonal array (as compared to conventional monopolar stimulation). In 2014, the same research group has verified the performance of virtual electrodes (Figure 2B (ii)) generated by steering the stimulation currents from two adjacent electrodes to the midpoints [115]. Such current steering techniques can be effective in stimulating retinal cells located at the midpoints of neighboring electrodes with a reasonable threshold level, contributing to higher effective channel density and thus to higher visual resolution. Subsequently, Dumm et al. evaluated the feasibility of virtual electrodes by current steering in retinal prostheses by varying the ratio of the stimulation current levels between two electrodes and recording the spatial shift of cortical activation patterns evoked by retinal stimulation [118]. Since 2016, this group has utilized the hexagonal configuration for current focusing and steering [119,120,121]. The hexagonal electrode array was supra-choroidally implanted in six normally sighted cats for multipolar stimulation to show not only lowered average voltage levels around the stimulating electrode, but also more focused cortical response patterns than when monopolar stimulation was used [119]. In 2018, the group used the similar electrode array implanted into seven normally sighted cats demonstrating focused stimulation and two-dimensional generation of virtual electrodes using current steering across multiple electrodes [120]. Cortical responses recorded from the cats suggested the effectiveness of current steering among up to six electrodes, which could increase the degree of visual resolution without increasing the number of physical electrodes. However, this strategy applied in the blind retina has shown insufficient steering effects compared to the healthy retina, indicating current steering may need further optimization for use in a degenerated retina [121].

#### 2.3.3. Optimization of Stimulation Parameters

Various ES parameters have been studied to improve the temporal and spatial resolution of electrically evoked retinal responses. Selective activation of specific retinal cell types has been demonstrated via optimization of stimulation waveforms. RGCs have been shown to be more affected by short pulse durations of less than 0.15 ms, whereas presynaptic neurons (e.g., bipolar cells, amacrine cells, and photoreceptors) have been more responsive to the pulses longer than 1 ms in duration [122,123]. Sinusoidal stimulation at 25 Hz and at 100 Hz could selectively activate bipolar cells and RGCs, respectively, implying that conventional rectangular pulses are likely to activate both types of cells [124]. In 2012, Nanduri et al. reported that the frequency modulation of biphasic rectangular pulses between 13 and 120 Hz better aided the achievement of higher resolution than conventional amplitude modulation. In the test on a human subject implanted with a 16-channel retinal electrode array, frequency-modulated stimulation produced phosphenes of higher contrast and smaller size [125]. The subsequent in vitro study in 2015 by Weitz et al. showed that sinusoidal stimulation for a long duration (25 ms) could selectively stimulate inner retinal neurons (either directly or indirectly) while avoiding activation of RGC axons. This stimulation could form 375-μm-high letter patterns on the human retina, corresponding to a Snellen visual acuity of 20/312 [126]. Similar in vitro results were presented in 2018, i.e., sinusoidal stimuli of a long duration (10 ms) could selectively stimulate the ON RGCs [127]. In 2019, another study reported that amplitude levels between 30 and 40 μA maximized the responses of the ON RGCs [128].

## 3. Optical Stimulation (OS)

The eye is a highly sophisticated optical system. It adaptively focuses light onto the retina using a series of optical elements including the cornea, iris, lens, and vitreous humor. The optically transparent structure of the eye renders optical stimulation (OS) a suitable modality for restoring retinal functions. OS can be more advantageous than ES for achieving higher spatial resolution, as light can be focused down to the diffraction limit, which is smaller than the respective diameters of cones (approximately 5 μm), bipolar cells (approximately 5 μm), and RGCs (approximately 15 μm), thereby potentially enabling modulation of cellular activities with single-cell resolution [129,130]. Despite its considerable potential, OS-based retinal prostheses remained undeveloped until the emergence of a novel optical neuromodulation technique, i.e., optogenetics. Optogenetics involves genetic modification of light-insensitive neurons so that they become sensitive to the light of certain wavelengths. Unlike the direct light sensitivity of normal cells, the neural activities of these neurons can additionally be modulated by electrical or chemical energy converted from incident optical energy, via nano- or micro-materials implanted near the neurons. In the following sections, various attempts to develop retinal prostheses based on optogenetics and exogenous materials implanted into retinal tissue will be discussed, with an emphasis on visual acuity and the stimulation methodology.

### 3.1. Classification of OS-Based Retinal Prostheses

There might be some ambiguity in categorization of OS-based retinal prostheses especially when the devices are employing both optical and electrical phenomena like photovoltaic prostheses. For the consistency of the categorization in the article, the retinal prostheses are categorized under OS approaches based on two criteria: (1) very first input applied to the eye is light and (2) the device is a stand-alone implant without any additional processing electronics or external power supply, mimicking the natural function of photoreceptors by directly generating stimulation in response to incident light. The OS-based retinal prostheses are then classified based on the types and the functionality of exogenous materials/structures applied to the retinal neurons. A summary of visual acuity from OS-based retinal prostheses is shown in Table 2. The OS-based retinal prostheses are then classified based on the types and the functionality of exogenous materials/structures applied to the retinal neurons. A summary of visual acuity from OS-based retinal prostheses is shown in Table 2.

#### 3.1.1. Optogenetics-Based Retinal Prostheses

Optogenetics is gene therapy applied for allowing cells to respond to light [131,132]. This technique has been adopted for visual prosthetics via transfection of foreign genes (e.g., a channel-rhodopsin, a halo-rhodopsin, and chemically engineered receptors expressing genes) into targeted retinal layers (e.g., RGCs, bipolar cells, and cones) to restore the light sensitivity of degenerated retinal cells. Optogenetics has gained increasing attention owing to its high spatial resolution, avoidance of invasive surgery, and ability to achieve both excitation and inhibition of neural activities [133,134,135,136].

Optogenetics-based retinal prostheses involve the injection of viral vectors containing microbial opsin genes into the target retina. Bi et al. demonstrated optogenetics-based visual restoration in retinal degenerated (rd)1/rd1 mice for the first time by expressing the channel-rhodopsin-2 (ChR2) at the RGCs [137]. Monitoring of the light-evoked electrical responses confirmed that light stimulation could successfully elicit action potentials from the dissociated retinal neuron. Visually evoked potentials (VEPs) were successfully elicited from the visual cortex when exposing ChR2-expressing rd1 mice to 460-nm light. Lagali et al. exploited the native processing schemes in the retinal network, by expressing the channel-rhodopsin at the ON bipolar cells [138]. Light-driven spikes from RGCs can be recorded using multi-electrode arrays (MEA), upon activation of the ChR2 in the ON bipolar cells. ChR2-activated VEPs monitored from the visual cortex of rd1 mice showed increased locomotor activities in the illuminated area, confirming that the activation of ChR2-expressed ON bipolar cells can induce behavioral changes in rd1 mice. For some cases where cones survived but lacked functionality, Busskamp et al. demonstrated that the expression of a halo-rhodopsin in the light-insensitive cones successfully restored the light sensitivity of the retina. In addition, the native phototransduction cascade was successfully substituted in the ex vivo human retina [4].

Unlike the optical activation of ion channels in optogenetics, G protein-coupled receptors (GPCRs) located in photoreceptors can be optically activated via high sensitivity to light involving signal amplification [139,140]. Though light-gated melanopsin GPCRs and rhodopsin GPCRs have been genetically introduced to RGCs and bipolar cells, respectively, it has been found that the ectopic expression of melanopsin and rhodopsin outside of the photoreceptors elicits slow light responses, limiting the visual restoration of fast-moving objects. To overcome this issue, Berry et al. introduced a nanoscopic chemical photoswitch for activating metabolic glutamate receptor 2 (mGluR2) to the RGCs; this approach elicited a rapid light response in the RGCs of isolated rd1 mice retinas, and a peak spike was attained in 355 ± 21 ms after the laser onset [141].

Despite their superior spatial resolution and non-invasiveness as compared to conventional ES-based retinal prostheses, optogenetics-based retinal prostheses present safety issues and technical challenges. The first challenge concerns the potential toxicity of viral vectors and ethical issues. Questions are being raised regarding the genetic modification of human genes for future clinical applications of optogenetics. A recent study has shown that the long-term expression of the ChR2 in rodent retinas did not cause any adverse side effects [142], but further safety data (particularly from non-human primates) are required. The damage of retinal neurons used to be the major drawback of the optogenetic approach as it requires intense light especially at the short wavelength near the ultraviolet (UV) range to activate retinal neurons. However, this is no longer the case with the recent development and use of more light-sensitive optogenetic tools such as the channel-rhodopsin using Ca^2+^ translocating channel-rhodopsin (CatCh) [143,144,145,146,147]. Other studies have demonstrated red-shifted opsins that can be activated by longer wavelengths, thereby avoiding potential light-induced tissue damage [148,149].

Some optogenetics-based studies on retinal prosthetics have evaluated the visual acuity of the artificial vision enabled. Berry et al. performed grating discrimination tests; mice with medium-wavelength (535 nm) cone opsin transfected in the RGCs could discern 0.056 cycles per degree (cpd). This was approximately a nine-fold reduction from the normal visual acuity of wild-type mice (0.3–0.5 cpd) [146]. Ganjawala et al. expressed chloromonas oogama (CoChR, a variant of channel-rhodopsin with improved light sensitivity) in the RGCs of mice and observed the highest visual acuity of 0.24 cpd [147]. Although the visual acuity of the optogenetic artificial vision in human subjects has not been evaluated so far, there are two independent ongoing human clinical trials in the United States and France, respectively, implying that the visual acuity would be tested in blind patients in the near future [150,151].

#### 3.1.2. Photovoltaic Retinal Prostheses

Photovoltaic retinal prostheses employ inorganic or organic microstructures implanted in the retina to convert incident light energy into electrical energy and trigger voltage-dependent ion channels for neural activation. This approach can avoid the concerns associated with genetic modification (e.g., in the optogenetic approach), while maintaining relatively good spatial resolution. Based on the criteria mentioned in 3.1., the Alpha IMS was categorized in ES-based retinal prostheses (Section 2) because even though the very first input applied to the eye is light, Alpha IMS generates stimulation pulses using built-in electronics and external power. This section will discuss other photovoltaic approaches that meet both the criteria.

The artificial silicon retina (ASR), from Optobionics in the United States, is the first sub-retinal prosthesis that has entered clinical trials [152,153]. The ASR is an inorganic silicon-based passive device that is only driven by incident light power. The device consists of approximately 5000 solar cells with a separation of 5 μm and each cell consisting of a 20 μm × 20 μm micro-photodiode and a 9 μm × 9 μm stimulating electrode. In clinical trials with 10 RP patients, the ASR was well-tolerated in the body, and improvements in the visual functions of the patients were observed [154]. However, the improvements primarily occurred in a location far from the implanted region. Subsequent investigations revealed that the improved vision was attributed to neuroprotective effects caused by retinal damage owing to the implantation of the ASR, rather than the electrical activation of the retinal cells by the ASR [155]. Additionally, the stimulation current generated from the photodiodes was found to be far below the micro-ampere level required for neural activation [116].

Another type of photovoltaic retinal prostheses not requiring an external power supply based on an inorganic material was demonstrated by Mathieson et al. [156]. A sub-retinally implanted silicon photodiode array converted pulsed near-infrared (NIR) patterns projected on the array into a local electric current to stimulate retinal neurons. The NIR patterns were generated by capturing images from a camera and projecting the translated NIR images via video goggles onto the photovoltaic implant. By exploiting the relatively high sensitivity of silicon in the NIR region and the three serially connected diodes, this device merely occupied a 0.8 mm × 1.2 mm silicon chip in a Royal College of Surgeons (RCS) rat retina [156]. The functionality of this photovoltaic retinal prosthesis has been assessed in RCS rats via confirmation that the NIR projection on photodiode-elicited cortical responses are similar to those evoked by visible light stimulation [157]. A subsequent study demonstrated that 70-µm-wide pixels consisting of two or three serially connected photodiodes produced receptive fields of 248 ± 59 µm in the RGCs, comparable to the 244 ± 32 µm fields elicited by visible light stimulation. RCS rats implanted by the device could distinguish black-and-white grating stripes with a width of 64 ± 11 µm (0.47 cpd), whereas wild-type rats could distinguish between stripes of 27 ± 9 µm width (1.1 cpd) [158]. After this silicon photodiode array-based device was acquired by the Pixium Vision, the device was named PRIMA and has been implanted in five patients for clinical trials. The outcomes showed three of the patients demonstrated the optimal visual acuity of 20/460 to 20/550 [159]. Silicon also can be used as a nanowire array for a high-resolution sub-retinal neurostimulator that directly transduces incident light into electrical stimulation [160]. In 2018, Nanovision demonstrated an array with 1512 nanowires addressed by IR light, which were functionally evaluated by eliciting cortical responses from the stimulation [161]. It is presently in the preclinical stage. Recently, a new type of an inorganic material has been proposed for photovoltaic retinal stimulation. It employs gold nanoparticles decorated with titania (Au-TiO_2_) nanowire arrays [162]. After sub-retinal implantation of the Au-TiO_2_ nanowire arrays in the isolated retinas of rd1/cone diphtheria toxin subunit-A (cDTA) blind mice, an optical spectrum in the range of near-UV, blue, and green was found to activate the RGCs, with a spatial resolution of 100 µm. Thus, light stimulation after sub-retinal implantation of the nanowires could reproduce VEPs at the primary visual cortex.

Photovoltaic retinal prostheses using organic materials have also gained attention, owing to their tissue-friendly properties, such as conformability and biocompatibility. Ghezzi et al. demonstrated that RGCs lying on a poly(3-hexylthiophene) (P3HT) organic film could be activated by ambient light illumination (wide-band light-emitting diode (LED) having peak at 532 nm) [163]. The daylight irradiance on the retina (0.1–10 µW/cm^2^) within the dynamic range of P3HT (1–100 µW/cm^2^) elucidated the potential retinal modulation capability using ambient light, without any external power supply. In subsequent studies, the P3HT layers sub-retinally implanted in the RCS rats restored some visual sensations. Monitoring of the VEPs at the binocular region of the primary visual cortex verified restored visual perception, with a visual acuity of 0.52 cpd after one month of implantation. Despite a subsequent slight decrease of the visual acuity to 0.38 cpd after six months, this prosthetic device generally maintains its functionality for up to 6–10 months after implantation [164].

#### 3.1.3. Photoswitch-Based Retinal Prostheses

Synthetic photoisomerizable and photoswitchable small molecules attached at the potassium channels of neurons have been shown to modulate neural activities, based on light-triggered conformational changes of the molecules [165]. Fortin et al. attached acrylamide-azobenzene-quaternary ammonium (AAQ) molecule as a photoswitchable affinity label to the cytoplasmic sides of potassium ion channels of rat RGCs [165]. Conversion of the AAQ shape from *cis*-form to *trans*-form upon illumination with a long wavelength (500 nm) blocked the potassium channels, thereby decreasing the excitability of the neurons. In contrast, AAQ changed to the *cis*-form at a short wavelength (380 nm), thereby unblocking the channels and increasing the neural activities [166]. Intraocular injection of AAQ enabled robust light-induced responses in the RGCs of genetically blind mice [166]. The visual acuity was determined in terms of the spatial extent of the RGC activities, defined as the receptive field of the RGCs. The RGCs responses were recorded using 60-channel MEA with 200-µm spacing, whereas the AAQ-treated retinas from the rd1 mice were illuminated with a light ray having a wavelength and a diameter of 380 nm and 60 µm, respectively. Light-evoked responses were consistently recorded from only a single electrode among the 60 electrodes, implying that the estimated receptive field of the RGCs is smaller than the inter-channel spacing of the MEA (200 µm) [166]. This technique presents an important advantage of being able to control neural excitability without involving any genetic manipulation. One limitation, however, is the necessity for near-UV light illumination, which may damage the retinal tissue and limit the penetration depth of the light into tissue [167].

#### 3.1.4. Photothermal Retinal Prostheses

Another modality of OS-based retinal stimulation is to use photothermal retinal prostheses where no exogenous material is administered to the eye. IR laser stimulation at a wavelength of 1875 nm was irradiated on RGCs of mice ex vivo with whole cell patch-clamp recording for evaluation of the transient membrane potential and temperature changes [168]. The results showed that thermosensitive transient receptor potential vanilloid (TRPV) 4 channels of the retina mediate the IR laser-evoked responses, thereby suggesting the potential of photothermal stimulation to facilitate the development of future prosthetic devices for blind patients.

### 3.2. Visual Acuity and Stimulation Methodology

The maximum numerical aperture (*NA*) of human eye is 0.23 when a diameter of the iris is set to 8 mm [169]. The resolution determined by the Rayleigh diffraction limit (*R*) can be given as follows:

*R* = 0.61*λ*/*NA*, (1)

where *λ* is the wavelength of the light. Based on Equation (1), light can be theoretically focused down to a dimension as small as 1.0 µm for 380-nm AAQ photoswitch-based activation and 1.4 µm for 532-nm halo-rhodopsin activation [132,165]. These values suggest that OS has the potential ability to enable neuromodulation at single-cell or even subcellular spatial resolution.

As the visual information generated in photoreceptors undergoes complex signal processing while passing through the multiple cell layers in the retina, the “image inputs” arriving at each retinal cell layer are all different, as a result of the “encoding” in the preceding layers. As the exact information processing functions of the retina, unfortunately, are not yet fully understood, it is quite complicated to determine the exact stimulation patterns that should be provided as inputs to each layer. The inputs should be different for each case in retinal prosthetic approaches, with different electrode placements and targeted cell types. Special stimulation patterns thus have been proposed for specific retinal cell types in light-sensitized cones, bipolar cells, and RGCs. For example, for the stimulation of cones, anisotropic filtering for image simplification and spatial image compression for fitting the image into the field of view were sequentially applied. For bipolar cell stimulation, spatiotemporal derivative maps were created, and the corresponding positive and negative values were applied to ON and OFF bipolar cell stimulation, respectively. Finally, a spike-coded map from the spatiotemporal derivatives was employed for the stimulation of the RGCs [170].

Despite largely unknown mechanisms of retinal signal processing, RGCs have been targeted for stimulation as they are known to be the most stable retinal neurons in the retinal remodeling process during which the existing retinal neurons are reorganized after degeneration of photoreceptors, resulting in ectopic synapse of survived cone cells, dendrite reduction, mislocation, and displacement of bipolar cells [171]. On the other hand, it has also been considered that the activation of presynaptic neurons such as bipolar cells and/or photoreceptors is more effective in terms of generating more naturalistic vision by exploiting the innate retinal network. Recent studies have shown indirect activation of RGCs by electrically stimulating photoreceptors or bipolar cells that has induced ON RGCs responses that have strong correlation to visually evoked ON RGCs responses, but not OFF RGCs [172,173]. This results support the hypothesis that activation of distal neurons could be effective in achieving fine vision restoration.

The spatial extent of neural responses to photovoltaic stimulation is limited by the size of the electrodes and current spreading, both of which in turn limit the resolution of artificial vision. The size of the electrodes determines the number of neural cells recruited by the stimulation, whereas the spreading of the stimulation current in the conductive neural tissue creates inter-channel interference. Optogenetics- and photoswitch-based approaches theoretically allow for neuromodulation with cellular-level resolution. It should be noted that a fair comparison of visual acuity of different studies summarized in Table 2 can be complicated as experimental settings, conditions and subjects are not identical. For instance, differences in native visual functions among species, e.g., the higher visual acuity of normal humans than rodents, may hinder a direct comparison of the visual acuity achieved by various retinal prosthetic devices. Therefore, it is recommended to consider all the experimental conditions of those studies for fair comparison.

**Table 2:** Optical stimulation (OS)-based retinal prostheses and their visual acuity.

Methodology	Species	Stimulation Target	Visual Acuity	Ref.
Optogenetics (medium-wavelength cone-opsin)	Mouse	RGCs (in vivo)	0.056 cpd	[146]
Optogenetics (CoChR)	Mouse	RGCs (in vivo)	0.24 cpd	[147]
Photovoltaic - inorganic (IR light-sensitive photodiode)	Rat	Bipolar cells (in vivo)	0.47 cpd, 20/460	[158,159]
Photovoltaic - organic (ambient light-sensitive photodiode)	Rat	Bipolar cells (in vivo)	0.62 cpd	[164]
Photoswitch (AAQ)	Mouse	RGCs (ex vivo)	<200 µm (receptive field of RGCs)	[166]

## 4. Ultrasonic Stimulation (US)

For the past 70 years, focused ultrasound has been extensively investigated for non-invasive neural stimulation [174,175,176,177]. As an example of ultrasonic stimulation (US), Tufail et al. demonstrated that focused ultrasonic pulses effectively modulate mammalian cortical and hippocampal activities [178,179]. More recently, focused ultrasound has been widely used for transcranial stimulation in the treatment of motor disorders [180,181]. Although the exact biophysical mechanisms underlying US remain largely unknown, some potential physical principles may include the activation of mechano-sensitive channels, generation of capacitive currents owing to membrane displacements, and opening of pores in the lipid bilayer [179].

A summary of the focused ultrasound approach applied for retinal stimulation is presented in Table 3. The first US-based retinal prosthesis by Naor et al. in 2012 elicited electrical activities in the retinal neurons of Sprague Dawley rats with normal vision by using ultrasonic waves at the acoustic frequencies of 0.5 MHz and 1 MHz [182,183]. Furthermore, the device could enable multi-focal US within a large visual angle, suggesting a resolution of 0.40–0.53 mm. Images captured by an external camera were acoustically projected onto the retina using an ultrasonic phased array placed on the cornea. In 2013, Menz et al. demonstrated stable responses elicited by US at 43 MHz in the isolated retina of tiger salamanders, whereby a spatial resolution of approximately 0.10 mm was achieved [184]. In this research, it has also been found that the US primarily activated the inter-neurons, rather than directly affecting the RGCs. In 2014, Wu et al. developed a front-end integrated circuit (IC) for non-invasive retinal stimulation using US at 40 MHz [185]. The proposed system could efficiently compute the US stimulation patterns from a two-dimensional transducer array using a fast Fourier transform algorithm embedded in the IC. Since 2017, a research group in China has investigated new types of non-invasive ultrasonic retinal prostheses based on a transducer array integrated into a contact lens, for easy attachment and enhanced acoustic coupling with the eye fluid [186,187,188]. The array can simultaneously generate multiple US focal points on two-dimensional surfaces. Its neurophysiological feasibility was demonstrated in vitro upon confirmation of the reliable activation of RGCs in response to focused low-frequency US [187]. In 2019, the same group demonstrated a 512-channel transducer array integrated in a ring-shaped arrangement on a contact lens. The array configuration was optimized via finite element analysis, and the results suggested an expected spatial resolution of approximately 0.60 mm [188].

## 5. Magnetic Stimulation

Magnetic stimulation (MS) is a fundamentally electrical phenomenon, but it can be enabled without any electrical contacts [189]. A powerful application based on MS is trans-cranial magnetic stimulation (TMS). TMS targets the cerebral cortex, spinal nerves, and peripheral nerves [190,191,192]. It provides clinically useful diagnostic and prognostic tools; moreover, it provides avenues for therapeutic applications of the treatments for neurological and psychiatric disorders. In regard to the visual system, TMS has been found to be capable of improving the retinal function in RCS rats with retinal degeneration [193].

Phosphenes evoked via direct MS on the retina were first observed in the 19th century, as reported by d’Arsonval [194]. In 1981, Lövsund et al. reported that magnetic fields could successfully evoke RGC responses in frog retinas. They also found that MS at 20 Hz produced the most sensitive responses of RGCs, based on a 20-mT threshold [195]. Similarly, the feasibility of MS for eliciting retinal responses was also verified by Shin et al., who used time-varying magnetic fields to generate eddy currents to stimulate the retina [196]. In 2005, the first MS-based epi-retinal prosthesis was proposed by Basham et al. through in vitro stimulation of the abdominal nerves of a crawfish. Its feasibility as another retinal stimulation modality has been demonstrated using ferrite cores to focus the magnetic flux within a small area on the retina for localized activation [197,198,199]. In 2012, Lee et al. reported that sub-millimeter micro-coils with dimensions of 1000 μm × 500 μm × 350 μm could evoke different retinal responses depending on the spatial orientation of the coils relative to the neurons, and these could be utilized for the selective activation of different retinal cells [200,201]. A following study in 2016 presented new single-loop micro-coils for inducing activation of the cortical neurons and behavioral responses in mice [202]. Although the effectiveness was shown in the cortex, the above test results suggest that the MS can create more focused activation of the retina than ES, and it can be exploited for visual prostheses that generally require highly precise activations of neurons [202].

## 6. Other New Approaches

Despite not discussed in detail, we also note a set of emerging technologies in the field of retinal prosthesis including chemical stimulation of the retina for higher resolution. In 2010, Finlayson and Iezzi presented chemical stimulation of RGCs using glutamate, which is the primary neurotransmitter released by photoreceptors, bipolar cells, and RGCs [203]. They hypothesized that the existing limitations of retinal prostheses can be circumvented by using more naturalistic means of stimulation in the retinal network. Glutamate locally applied via micropipettes on RGCs of both normal and photoreceptor-degenerated rats could effectively excite the RGCs of both rats. The duration of the retinal activity was highly dependent on the duration of the glutamate application. Subsequently, Inayat et al. in 2014 also investigated the glutamate-based chemical stimulation to demonstrate that the spatial spread of the stimulation was approximately 290 μm from the injection site, which was comparable to the current electrical prostheses [204]. These results indicate the feasibility of neurotransmitter-based chemical stimulation as a viable alternative of ES.7. Conclusions and Outlook.

**Figure 3:**
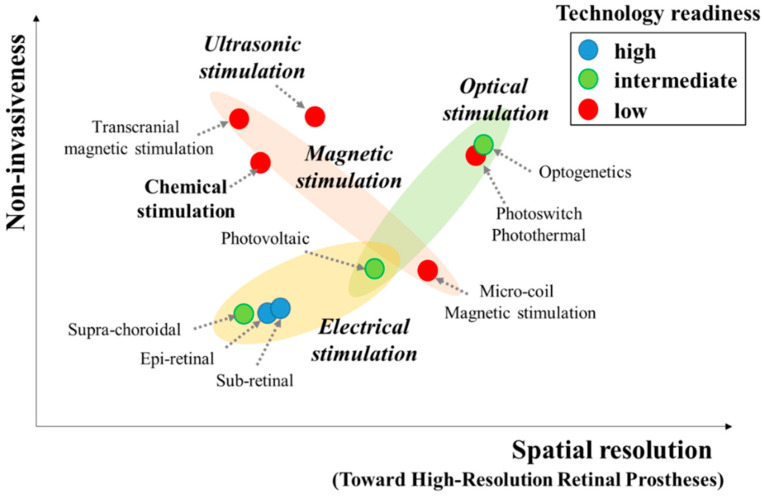
A two-dimensional representation comparing spatial resolution and invasiveness of various retinal prosthetic approaches discussed in this article, along with color-coded description on technology readiness.

Undoubtedly, retinal prostheses can offer enormous hope to blind patients who suffer from RP and AMD and cannot be cured using existing pharmacological or surgical treatments. Many efforts have been made to deliver promising results, from both engineering and clinical perspectives. However, retinal prostheses have not yet been able to provide sufficient visual capabilities for visually impaired patients to experience everyday life independently, mainly owing to the low spatial resolution of the restored vision. In this review article, the endeavors toward achieving enhanced visual perception have been addressed from many standpoints, e.g., the electrode structures, stimulation parameters, and stimulation modalities. Figure 3 illustrates a two-dimensional representation for comparing spatial resolution, invasiveness, and technological readiness of various retinal stimulation modalities toward high-resolution retinal prostheses.

The most successful retinal prostheses developed to date are based on ES. The feasibility of vision restoration has been demonstrated even for end-stage RP patients, whereby they have been able to perform real-world functional tasks including phosphene localization, letter reading, and object detection. The best visual acuity (20/312) was expected from long-duration sinusoidal pulses, which can selectively stimulate inner retinal neurons. Extensive efforts, nevertheless, will be required to overcome the limitations regarding the spatial resolution of artificial vision, which is currently lower than the theoretically estimated resolution. The major issues to be overcome include inefficient electrode contacts to retinal neurons, electric currents spreading in conductive retinal tissues, and differences in neural responses to ES and natural vision.

The OS-based approaches have gained increasing attention, as they could potentially overcome the limitations of ES-based approaches. Proof-of-concept studies have shown encouraging results in eliciting visual perception in animal models. Utilization of the native optical functions of the eyes in the retinal prosthesis for projecting images onto the retina could minimize the surgical procedures required for the OS-based retinal prosthesis. Moreover, OS is potentially advantageous for higher spatial resolution, as light can be focused down to a theoretical diffraction limit smaller than the cell diameters, enabling neural activation at a single-cell resolution.

US, at present, prompts several fundamental questions to be addressed before it can be practically applied, including the exact biophysical mechanisms of US and the lack of demonstrated visual acuity data from multi-focal US stimulation. Nevertheless, US has also been considered as a suitable candidate, owing to its non-invasiveness and potential suitability for high-resolution stimulation. The highest spatial resolution using US was shown in that 0.10 mm-sized features could be distinguished with an acoustic frequency of 43 MHz. US with a higher frequency is expected to achieve a finer resolution.

Finally, while MS is in a relatively early stage as compared to other approaches, the highly focused and precise retinal activation demonstrated by MS is encouraging. Further studies are required mainly to provide multi-point stimulation for creating complex stimulation patterns.

Despite all the promising results summarized in this article, there remain many hurdles to be resolved to enable successful restoration of vision. For example, not discussed in this article focusing on the visual acuity enhancement, proximate interfacing with the retinal tissue is another important issue of current retinal prostheses, thus newer materials such as conducting polymers, carbon nanotubes, and graphene oxide have been recently explored [205,206,207,208,209]. Future endeavors are expected to overcome the existing technological and physiological limitations. Continued advances in both engineering and physiology for novel technological solutions (as well as for understanding retinal functions) will promote the development of next-generation retinal prostheses, potentially offering blind patients visual perception comparable to that of normal vision.

## Figures and Tables

**Table 1:** A summary of three approaches to current retinal prostheses applied in clinical trials.

Type	Device Name	Electrodes			Clinical Trial Results			Ref.
		Number	Size (μm)	Pitch (μm)	Number of Subjects	Visual Field	Visual Acuity ^8^	
Epi-retinal	Argus II	60	Ø 200	525	30	~20°	20/1262 (grating)	[67,68]
	IMI ^1^	49	Ø 250	120	20 ^6^, 7 ^7^	-	-	[69,70,71,72]
	IRIS ^2^	150	-	-	20	-	-	[73]
	EPI-RET3	25	Ø 100 ^5^	500	6	-	-	[35,74]
Sub-retinal	Alpha IMS	1500	50 × 50	70	29	11° × 11°	20/546 (Landolt-C)	[37,66]
	Alpha AMS	1600	-	-	15	~15°	-	[75,76]
Supra-choroidal	BVA ^3^	33	Ø 400, Ø 600	1000	3	~12°	20/4451 (Landolt-C)	[77]
	STS ^4^	49	Ø 500	700	2	20° × 16°	-	[36]

^1^ Intelligent Medical Implants; ^2^ Intelligent Retinal Implant System; ^3^ Bionic Vision Australia; ^4^ Semichronic Suprachoroid Transscleral; ^5^ With a height of 25 μm; ^6^ Acute; ^7^ Chronic; ^8^ A measure of the spatial resolution of the visual system.

**Table 3:** A summary of prosthetic uses of focused ultrasound in retinal stimulation.

Transducer	Device Placement	Stimulation Target	Acoustic Frequency (MHz)	Average Intensity (W/cm^2^)	Spatial Resolution (mm)	Ref.
Phased array	External to the cornea ^2^	RGCs ^3^ (simulation, in vivo)	0.5	0.12–0.42	0.40–0.53 (estimated)	[182,183]
			1.0	5.15–8.52		
Single transducer	-	RGCs ^4^ (in vitro)	43	10.0–30.0	~0.10	[184]
2D CMUT ^1^	In front of the eye	Retina (simulation)	40	-	-	[185]
Racing array	Attached on the cornea like a contact lens	RGCs ^5^ (simulation)	2.5	0.20–0.60	1.30	[186,188]
			5.0		0.60	
			10		0.26	

^1^ Two-dimensional capacitive micromachined ultrasonic transducer; ^2^ An acoustic coupling component was used, such as a bag of water or coupling gel; ^3^ Sprague Dawley rats were used; ^4^ The isolated retina of a tiger salamander was used; ^5^ A 5 MHz racing array transducer was mainly simulated to optimize the array configuration.
